# Diphallia with Associated Anomalies: A Case Report and Literature Review

**DOI:** 10.1155/2013/192960

**Published:** 2013-12-08

**Authors:** Pande Made Wisnu Tirtayasa, Robertus Bebet Prasetyo, Arry Rodjani

**Affiliations:** ^1^Department of Urology, Cipto Mangunkusumo Hospital, Jl. Diponegoro No. 71, Jakarta 10430, Indonesia; ^2^Department of Surgery, Division of Urology, Gatot Subroto Army Hospital, Jakarta 10410, Indonesia

## Abstract

Diphallia or penile duplication is an extremely rare congenital anomaly. It occurs once in every 5.5 million live births. The extent of penile duplication and the number of associated anomalies vary greatly, ranging from a double glans from a penis with no associated anomaly up to complete penile duplication associated with multiple anomalies. Here, we report a 12-year-old boy with complete bifid diphallia associated with bifid scrotum, epispadia, and pubic symphysis diastasis along with a review of the articles pertaining to this anomaly.

## 1. Introduction

Diphallia or penile duplication is an extremely rare congenital anomaly. It occurs once in every 5.5 million live births [1–4]. The first case was reported in 1609 by Wecker in Bologna, Italy [4, 5]. Until today, only about 100 cases are reported in the literature [4–6]. In Indonesia, this case was never reported. We report a case of bifid diphallia associated with bifid scrotum, epispadia, and pubic symphysis diastasis.

## 2. Case Report

A 12-year-old boy was referred as a case of penile duplication and bifid scrotum. The patient belonged to a low socioeconomic stratum. His complaint was unable to hold to urinate and the urine was always leaking from both of his penises.

**Figure 1:**
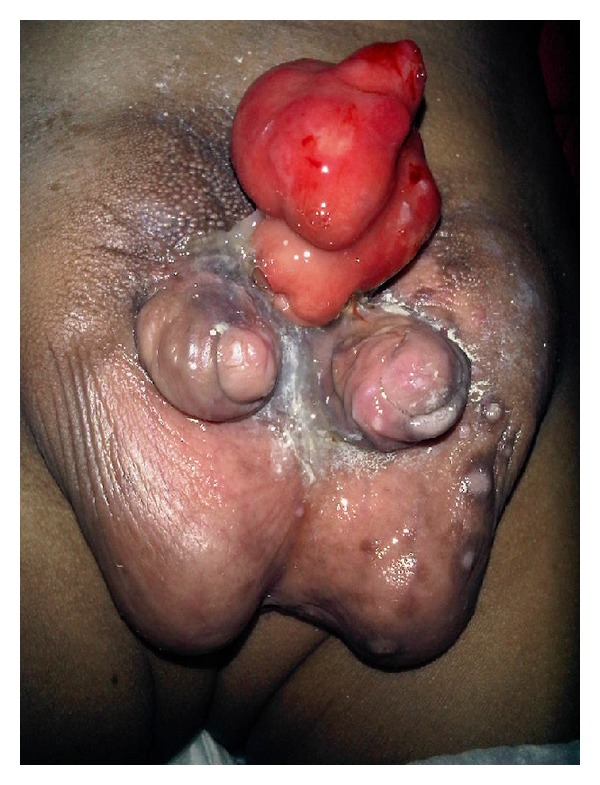
Complete and separate diphallia. Over the region of the pubis, a bowel loop-like structure was seen.

Examination of the genitalia revealed complete and separate penises of equal size, each with an epispadia urethral meatus. The scrotum was bifid, and each side contained a testicle. Over the region of the pubis, a bowel loop-like structure was seen (Figure 1). It was neither reducible nor had any relation with the rise in intra-abdominal pressure, apparently having no communication with the intra-abdominal contents.

Both urethral orifices were catheterized easily and ended up in a single bladder. On urethrocystoscopy done on the left penis, the bladder neck was directly seen without urethral mucosa prior to it. The bladder seemed hyperemic. On the right penis, there was fibrotic tissue along the urethra to the bladder. Retrograde pyelography revealed normal ureter and no vesicoureteral reflux. Plain photo showed normal vertebrae with pubic symphysis diastasis (Figure 3).

**Figure 2:**
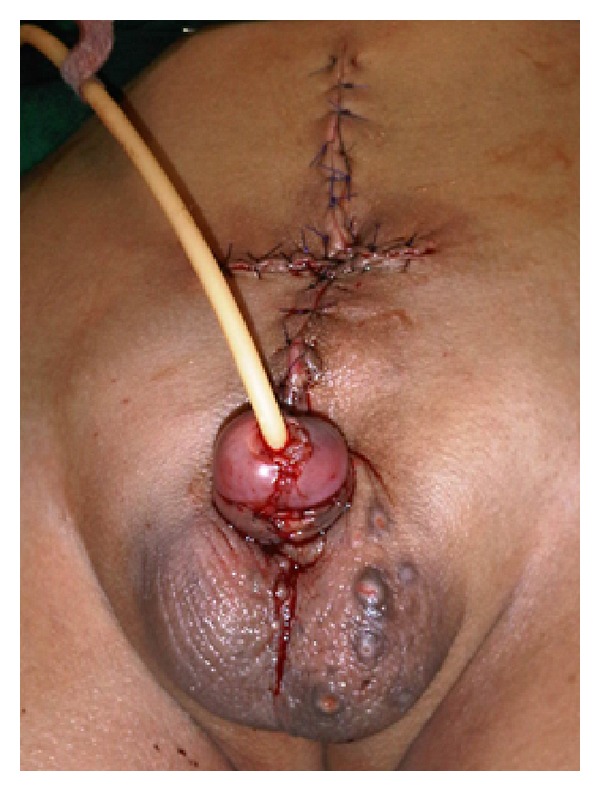
Final result after surgery.

**Figure 3:**
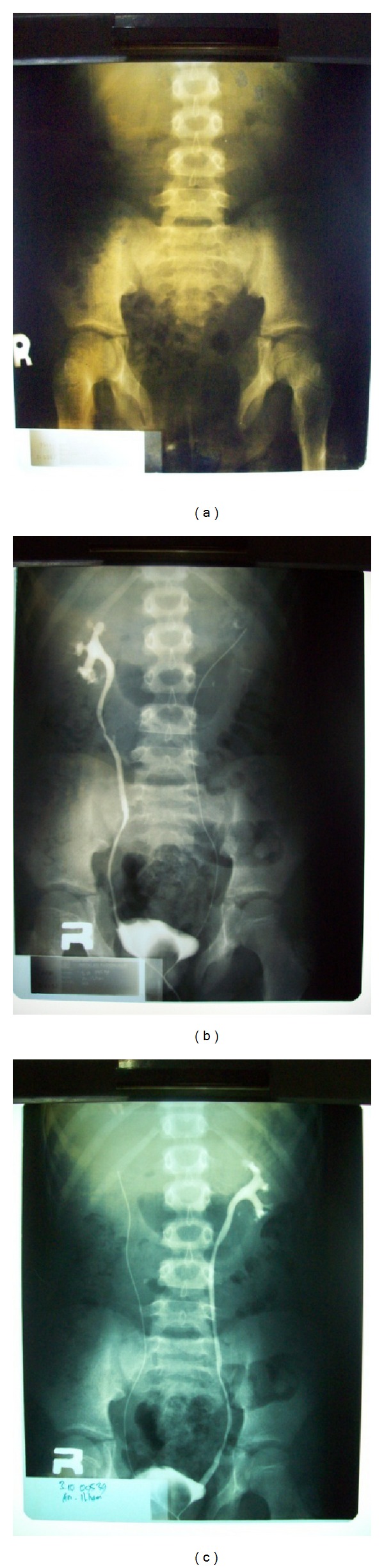
Plain photo showed pubic symphysis diastasis (a); retrograde pyelography showed normal ureter and no ureterovesical reflux (b) and (c).

At surgery, the exteriorized intestinal loop-like structure was excised. The structure had no communication with neither the peritoneal cavity and bladder nor other structures. Each penis was found to have only one corpus cavernosum. Epispadia repair and penis reconstruction were done by joining the corporal bodies in each penis (Figure 2). Biopsy revealed an intestinal mucosal pattern suggestive of colonic tissue. The patient's postoperative period was uneventful. Next plan was to correct the pubic symphysis diastasis by bone grafting.

## 3. Discussion and Literature Review

Diphallia is a rare anomaly, and it is believed that no cases are identical. It varies from a small accessory penis or duplication of the glans to complete penile duplication. The duplication may be orthotopic or ectopic. Division of the penis may be sagittal or frontal and symmetric or asymmetric, in shape and size [1, 3]. Schneider divided diphallia into three groups [2–4]: diphallia of the glans alone, bifid diphallia, and complete diphallia. Villanova and Raventos added a fourth category, pseudodiphallia. The urethra shows a range of variations, from functioning double urethras to complete absence of the urethra in each penis [2]. The majority have a single corpus cavernosum in each organ. The meatus may be normal, hypospadia, or epispadia, and the scrotum may be normal or bifid. The testes are normal, athropic, or undescended [2, 4]. A later classification currently widely accepted includes two main groups: true diphallia and bifid phallus [3, 4]. These two groups are further divided into partial or complete duplication. True complete diphallia refers to complete penile duplication, each with two corpora cavernosa and a corpus spongiosum. When the duplicate penis is smaller or rudimentary, it corresponds to true partial diphallia. When only one corpus cavernosum is present in each penis, the term bifid phallus applies. When the degree of separation is complete to the base of the shaft or to just the glans, the anomaly is considered complete or partial bifid phallus, respectively. The term “pseudodiphallia,” as originally described by Villanova and Raventos, corresponds to true, partial diphallia [3, 5]. Our case is a complete bifid phallus.

Diphallia is usually associated with other malformations, such as bladder and urethra duplication, exstrophy of the cloaca, exstrophy of the bladder, anorectal malformation, colon and rectosigmoid duplication, ventral hernia, pubic symphysis diastasis, abnormality of skeletal and heart muscles, and vertebral anomalies [1–5, 7–17]. True diphallia is more often associated with severe malformations compared with bifid phallus [3, 18]. Infants born with diphallia and its related conditions have higher death rate from various infections associated with their more complex renal or colorectal systems [19]. In cases of bifid phallus and orthotopic, true, and complete diphallia, both phalli are attached to the pubic bone; otherwise in the cases of pseudodiphallia or true, partial diphallia with an ectopic penis, the corpora are usually not attached to the pubis. In this case, amputation of the accessory penis is easier [3, 20].

The degree of erectile function in cases of diphallia varies significantly. Usually, one or both penises are capable of erection. In cases of true, complete diphallia presented at advanced age, simultaneous erection and, occasionally, ejaculation have been reported. In contrast, pseudodiphallia is rarely associated with normal function of the rudimentary phallus. Several studies reported normal erection of partial bifid phallus and true partial diphallia even though erectile function after surgery is still unclear [3, 7].

Diphallia is believed to take place during the embryonic development of the penis around the 3rd to 6th week of gestation [5]. In contrast, many authors have accepted that anomalies take place in the process of migration ventrally and fusion of the paired mesodermal anlagen by the 15th week of gestation [4]. The embryologic explanation of diphallia is quite obscure, because at no time during normal development is the genital tubercle a paired structure. Historically, duplication of the penis and bladder has been considered the end result of incompletely fused bilateral anlagen or a form of atavism, because reptiles such as snakes and lizards possess a double penis. Moreover, diphallia could represent a teratoid structure or a form of incomplete twinning. Still, none of these explanations is adequate and the wide spectrum of the anomalies cannot be explained by a single hypothesis [1–3, 21]. Karyotype in cases of diphallia has been found to be normal, with the exception of a case described by Karna and Kapur, which was associated with a balance translocation. The later suggested that defects in homeobox genes, which are thought to be master-controller genes of differentiation, may be involved in diphallia formation and related syndromes [15].

Detailed studies of the developmental anatomy of the genital tubercle have shed light onto the understanding of diphallia. The normal development of the penis begins with the coalescence of bilateral cloacal tubercles at the anterior end of the pars phallica of the urogenital sinus. Columns of mesoderm growing rapidly around the lateral margins of the cloacal plate form the genital tubercle. These bands of mesoderm arise from more than one area and their failure to fuse would lead expectantly to bladder exstrophy and epispadia with a split penis, instead of true diphallia. Consequently, another step is necessary for complete penile duplication to occur. An embryologic explanation of collateral urethral duplication includes the possibility of a longitudinal duplication of the cloacal membrane that would allow three or four columns of primitive streak mesoderm to migrate ventrally around the two cloacal membranes to eventually form two genital tubercles. Duplication of cloacal membrane in this fashion could also explain the frequent concomitant bladder, colon, anal, and spinal anomalies [2–5].

Penile duplication poses a difficult treatment problem in terms of medical, ethical, and aesthetic decision making. Detailed study of the external genitalia anatomy not only helps to classify the degree of penile duplication but also assists in the excision or reconstruction of the duplicate penis, by delineating the corporal development and urethral anatomy [3]. Ultrasound is used to help confirm the diagnosis. It detects the number of corpora cavernosum or corpora spongiosum and their accompanying abnormalities. Improved interpretation of anatomical structures has been made possible with the advent of magnetic resonance and better decisions can be made when carrying out surgical interventions [5, 18].

The treatment of diphallia is by excision of the duplicated noncommunicating penis. Excision of ectopic penis was reported in many bodies of literature [6, 15, 20, 22, 23]. Priyadarshi and Djordjevic et al. reported penis reconstruction by joining the corporal bodies in each penis in a patient with true complete diphallia [2, 17, 24].

Treatment principally depends on the type of accompanying congenital abnormalities as well as preserving continence and erectile function, which means individualizing each case [5, 23]. Surgical correction is individualized with the aims of achieving proper urinary continence, urinary stream, and erection with adequate cosmesis [1, 3, 5].
